# “I just get scared it’s going to happen again”: a qualitative study of the psychosocial impact of pediatric burns from the child’s perspective

**DOI:** 10.1186/s12887-023-04105-y

**Published:** 2023-06-05

**Authors:** Alix Woolard, Nicole Wickens, Lisa McGivern, Patricia de Gouveia Belinelo, Lisa Martin, Fiona Wood, Elmie Janse van Rensburg, Helen Milroy

**Affiliations:** 1 Telethon Kids Institute, Perth Children’s Hospital, 15 Hospital Avenue, Nedlands, Australia; 2 The University of Western Australia, 35 Stirling Highway, Crawley, WA 6009 Australia; 3 Burn Service of Western Australia, Fiona Stanley Hospital, MNH (B) Main Hospital, Level 4, Burns Unit, 102-118 Murdoch Drive, Murdoch, WA 6150 Australia; 4 Fiona Wood Foundation, 11 Robin Warren Drive, Murdoch, WA 6150 Australia

**Keywords:** Burn, Pediatric, Mental health, Psychosocial

## Abstract

**Background:**

Advances in medicine have improved the chances of survival following burn injuries, however, psychosocial outcomes have not seen the same improvement, and burn injuries can be distressing for both the child or young person, negatively affecting their wellbeing. Pediatric burn patients are at a higher risk of developing psychopathology compared to the general population. In order to promote resilience and prevent psychopathology post-burn injury for pediatric burn patients, it is crucial to understand the experience of children and young people after a burn. This study aimed to understand the psychosocial impact that a pediatric burn has as perceived by the pediatric burn patient.

**Methods:**

Seven pediatric burn patients were interviewed from the Perth Metropolitan area on average 3.1 years after their injury. All participants had been admitted to hospital for their acute injury and stayed for a median length of 2 days in hospital. Interviews with pediatric patients took place online, and the patients were asked about their mental health, coping strategies, changes to lifestyle and supports following their burn injury. The interviews were transcribed and then thematically analysed using an inductive approach.

**Results:**

Three overarching themes were developed from the interviews: burn-specific impact on the child or young person (including appearance concerns, family factors, and lifestyle factors), the psychological impact (including positive and negative impact on mental health), and factors supporting the recovery journey (including coping strategies and support services). The participants in our study highlighted issues they faced during recovery, the positive and negative impacts of the injury and recovery process and provided suggestions for future opportunities to bolster resilience and promote growth for pediatric burn patients who may face similar challenges in the future.

**Conclusion:**

Factors that improve the mental health and wellbeing of pediatric burn patients should be promoted, such as mental health and social supports, the promotion of adaptive coping mechanisms, and meeting the needs of the family unit as a whole. Ultimately, the implementation of trauma-focused, family centred interventions is crucial for the psychosocial recovery of pediatric burn survivors.

**Supplementary Information:**

The online version contains supplementary material available at 10.1186/s12887-023-04105-y.

## Introduction

Medical trauma is the psychological and physiological response to pain, injury, illness, medical procedures and treatments [1]. Burn injuries are a common medical trauma impacting children and young people. In Australia and New Zealand, children and young people under the age of 15 made up a third (30.9%) of hospital admissions relating to burns between 2009 and 2021 [2]. Advances in medicine have improved the chances of survival following larger burn injuries and thus patients might live with greater impacts of burn injury and more scarring [3]. In addition, better scar treatments have improved the physical health outcomes of those who have experienced a burn injury [4]. However, psychosocial outcomes have not seen the same improvement, and burn injuries can be distressing for both the child or young person, negatively affecting their wellbeing [5].

Children and young people who have experienced a burn injury (hereafter ‘pediatric burn patient’) are at a higher risk of developing psychopathology compared to the general population [5, 6]. Studies show pediatric burn patients are at risk of developing posttraumatic stress symptoms or Posttraumatic Stress Disorder (PTSD) within the first month after the burn injury [7–9]. Long-term psychological issues are also reported, with children and young people who have experienced a burn reported to experience elevated anxiety, depression, sleep disturbances, social functioning, and quality of life [5].

These statistics are not surprising given the complexity of burn aftercare, which often involves multiple invasive and painful treatments and procedures to improve function and lessen scarring [10]. Frequent hospital visits are also often reported to be a source of distress for pediatric burn patients, as it serves as a reminder of the injury [11]. The scarring following a burn can also be a source of distress for pediatric patients, particularly if the scar is visible [12–14].

Although pediatric burn patients face challenges post-burn, it is also clear that this population can experience resilience following their injury [15]. A recent review showed factors like social support, having purpose, and personality variables like extroversion, can contribute to resilience following a burn [15]. In order to promote resilience and prevent psychopathology post-burn injury for pediatric burn patients, it is crucial to understand the experience of children and young people after a burn. Further, community involvement in research is essential to ensure lived experience is considered when designing and implementing interventions and treatments [16]. To design trauma-informed, person-centred treatments for burn injuries, health professionals and researchers must understand the experience of the injury from the perspective of the child or young person themselves. Therefore, this study aimed to understand the psychosocial impact that a pediatric burn has as perceived by the pediatric burn patient.

## Method

### Participants

The current study reports on interviews with pediatric burn patient aged 12 years and older who were admitted with an acute burn injury to the Perth Children’s Hospital (PCH) outpatient burns treatment clinic in Western Australia. Only patients aged 12 years and older were eligible to participate in this study, given the emotional maturity required to discuss potentially distressing events such as their burn injury.

Parents were also invited to participate in interviews about their experiences, however the results of those interviews are not reported in this study but rather in an earlier publication [17]. Participants were excluded from the study if their burn was intentional or if they did not consent to take part in the study. Prior to the recruitment process commencing, the interviewers had no previous association with participants.

### Ethics approval and consent to participate

Ethics approval was obtained from the WA Child and Adolescent Health Service Human Research Ethics Committee (RGS4669), and informed written consent was provided by all participants including consent from legal guardians/parents and young people themselves. Participants were able to voluntarily withdraw from the study at any time without consequence or reason at any stage. All methods in this study were carried out in accordance with relevant guidelines and regulations specified by the Human Research Ethics Committee.

### Procedure

The staff from the burn treatment clinic provided the research team with the contact details of caregivers of eligible participants. Caregivers were contacted via telephone or email to invite their child to participate, and those interested were provided with further information about the study. If their child was willing to participate, they were asked to return a written consent form and a demographic form which enquired about the patient’s age, gender, residential suburb and whether they were diagnosed with any mental health conditions before or after the burn injury. Burn characteristics were obtained from the patient’s medical file provided by the clinic staff.

In total, 47 children/young people who had sustained a burn injury were eligible to participate in the interviews. Due to project delays, the research team could not contact 18 (38%) children/young people. Furthermore, one young person was uncontactable, 19 (40%) declined, and two (< 1%) children/young people agreed to participate but did not return consent forms.

Interviews were conducted online as semi-structured interviews between February and May 2022, using Microsoft Teams. All interviews were carried out by a provisionally registered psychologist and PhD candidate (EJ), except for one interview conducted by a registered general psychologist due to staffing changes. All participants assented to the interviews being digitally recorded and interviews lasted 43 min on average (mean) (range: 30–70 min). Before interviews commenced, participants were informed they could decline answering any uncomfortable questions or withdraw from the interview at any time. The interview guide (see Appendix 1) consisted of open-ended questions about general topics initially to build rapport, then participants were asked to describe their mental health since experiencing the burn injury, coping strategies, changes to their lifestyle, and sources of support. The guide was piloted with a 12-year-old burn survivor, followed by a workshop with the clinical and research teams to ensure appropriateness. There were no changes made to the interview guide following the above processes.

For context, this study is part of a larger research project called The Wellbeing Project, which aimed to (1) co-design a brief trauma-informed intervention to promote mental health recovery in children who have sustained a burn injury and (2) pilot the co-design intervention. In addition to exploring young people’s experiences following a burn injury, they were also given the opportunity to evaluate the proposed trauma-informed intervention. To facilitate this process, the intervention outline and resource materials were presented to participants, after which, they were asked a series of questions to provide feedback for the intervention. This feedback has not been included within the current study.

Participants were provided with a list of support services at the conclusion of the interviews, and interviewers referred any participants who indicated a need for additional psychological help or social support to the appropriate clinical psychological or medical services, as well as the PCH Burns Clinic Research Manager for follow-up. The PCH Burns Service provides immediate and ongoing treatment of burn injuries and related scarring. If paediatric burn patients at PCH indicate any sign of psychological distress, the clinical team recommend that they attend their general practitioner (GP) for a mental health assessment. However, mental health is often overlooked in post-burn recovery and issues may only arise once they have been discharged.

### Data analysis

All interview recordings were imported into the online transcription service Otter [18] and manually checked by one of the research team members to ensure accuracy (PDGB, NW and EJ). The transcripts were de-identified, and participant names were replaced with pseudonyms to ensure confidentiality. Interviews were then analysed using NVivo [19], which assisted in the coding process. Each interview was independently coded by at least two authors (PDGB, NW and EJ). Furthermore, three authors (PDGB, NW and EJ) analysed the interviews using Braun and Clarke’s reflexive thematic analysis [20–22], which involved familiarising themselves with the transcripts, semantically coding noteworthy pieces of text, then grouping codes into themes based on relevance in relation to each other. To fully adhere to reflexive thematic analysis and ensure rigour, these codes were triangulated and refined by four authors (PDGB, NW, EJ, and AW). In addition, two authors (AW, NW) documented reflective and developing ideas concerning the final themes and critical quotes during this iterative process.

## Results

A total of seven participants (five females and two males) aged between 12 and 18 years (mean 13.85, SD 2.27) were interviewed, the majority lived in the Perth Metropolitan area (n = 6, 86%; see Table 1). Participants were aged between 4 and 14 years at the time of the burn injury, and interviews were conducted on average 3.10 years after the burn injury. All participants had been admitted to the hospital for their acute burn injury with a median length of stay of 2 days. All participants had surgery for wound closure, and three participants had laser treatment for scar management, one of whom had also received serial reconstructive surgery and releases for the hand scar she sustained at a young age. Injuries were from flame, scald (i.e., liquid from the kettle, tap, saucepan, or food) or contact burn (i.e., a solid hot object such as BBQ, iron, or hot ash). Total burn surface area (TBSA) ranged from one to seven per cent. No participants reported being diagnosed with mental health conditions before or after their burn injury. However, two participants reported that their mental health had worsened since the burn (“increased general anxiety”, “increased anxiety/fear of the accident”).

**Table 1:** Participant demographics and burn characteristics

Characteristics of paediatric burn (*N* = 7)		
	***Median***	***Range***
**Age at burn injury (years)**	12.5	4.83–14.33
**Time since burn (years)**	1.92	0.33–13.42
**Total length of hospital stay (days)**	2	0.5–15
**TBSA (%)**	1.65	1–7
**Number of dress changes at**		
Acute admission	1	1
Surgery	1	0–1
Inpatient	1	0–3
Outpatient clinic	0	0–5
**Type of burn**	***n***	**%**
Contact	2	14
Scald	4	57
Flame	1	29

### Themes

Three overarching categories were identified during data analysis: burn-specific impact on the child or young person, the psychological impact of the burn injury on the child or young person, and factors supporting the recovery journey (see Table 2). Within these categories, key themes and sub-themes were generated. All direct quotes are presented in quotation marks and the following pseudonyms have been used to protect participant confidentiality: participant ID (#XX), gender (M/F), age at injury (X yo) in years, and time since injury (XXTSI).

**Table 2:** Interview categories, themes, and sub-themes

Categories	Themes	Sub-themes
Burn-specific impact on the child or young person	Appearance concerns	Scarring Garments coming off Response from other people
	Family factors	Family guilt around the injury Vicarious anxiety
	Lifestyle factors	Missing out/not being able to do the same activities Disrupted routine
Psychological impact of the burn injury on the child or young person	Negative impacts on mental health	Anxiety Fear Posttraumatic stress
	Positive impacts on mental health	Resilience
Factors supporting the recovery journey	Coping strategies	Acceptance Holistic approach Mindfulness Social support
	Support services	Hospital settings Counselling

### Burn-specific impact on the child or young person

#### Appearance concerns

scarring is common after a burn injury and participants described being self-conscious about how their scar would be perceived by others. One participant described her worries about having to explain her scar.

*“…what if I have to hold someone’s hand or something, and they don’t want to, and just knowing that you’re with kids that aren’t going to really understand what happened and make comments” (#31, F, 13yo, 13.42TSI)*

One participant explained that the pressure garment, used during the healing process of a burn, was protective when they wore it. However, once the garment came off, this was a source of distress as the scar became visible to others:

*“I had to stop wearing my garment, for me it was also hard to look at… I was kind of thinking ahead and saying …What if they [other people] judge me?” (#10, F, 12yo, 0.72TSI)*

A common experience for participants was the worry about how others would perceive them after the burn injury. Three participants described worrying about how their peers would view their scars. For example, one participant stated:

*“… the worry of having my burn on my hand and thinking that it looked scary, and being worried about how that would be received by my peers” (#31, F, 18yo, 13.42TSI)*

#### Family factors

participants were very cognisant of the guilt that their caregivers or family members felt about the burn injury. Three participants explained that they wished their caregivers were offered support to deal with the feelings of shame and guilt brought on by the burn injury. One participant who experienced a burn injury while staying at his grandparent’s house stated:

*“I had to convince my parents and grandad it wasn’t their fault. It was a bit frustrating because there was no way they could have affected the injury, my grandad felt really bad because he didn’t tell me that sparks could set cotton on fire” (#53, M, 16yo, 2.17TSI) *

Another participant acknowledged that their mother’s anxiety influenced their own mental health:

*“Her getting that kind of anxiety from that. And then that also kind of translating to me as well” (#31, F, 18yo, 13.42TSI)*

#### Lifestyle factors

participants often talked about how the burn injury caused disruption to their lifestyle and routine, such as missing out on sports or schooling, which in turn impacted their wellbeing. Some participants talked about how it took them a while to heal enough to be able to physically play sports. Others talked about how they were anxious to start playing sport again in case they injured themselves.

*“And whether it be even just mental, like being scared that something’s gonna hit my hand too hard. It’s a fear of mine, I guess. But I still do netball casually, but I can’t do serious netball anymore.” (#31, F, 18yo, 13.42TSI)*

All participants talked about missing school because of their injury. One participant summed up their experience:

*“…when I used to have all my appointments all the time, I’d always miss out on school” (#22, F, 12yo, 2.17TSI)*

### Psychological impact of the burn injury on the child or young person

#### Negative impact on mental health

many participants reflected on the hardships they faced after the burn injury, and how the injury itself had a negative impact on their mental health. Anxiety was the most commonly reported issue among participants. Participants talked about catastrophising thoughts (i.e., a cognitive distortion where the person thinks the worst outcome will happen), fear of fires or burn injuries occurring again, and feeling anxious following their injury. One participant stated.

*“Because I was exposed to that kind of stuff … making me yeah, just kind of anxious about the world around me and aware of quite heavy stuff when I couldn’t really process it in a healthy way” (#31, F, 18yo, 13.42TSI)*

Posttraumatic stress was also a clear theme, with participants describing symptoms of hypervigilance, repetitive thoughts about the injury, hallucinations, and in one case, nightmares. One participant described intrusive thoughts about the accident:

*“I just have that memory… and then I just get a bit more scared that’s going to happen again” (#10, F, 12yo, 0.72TSI)*

One participant described the low mood they experienced after their injury:

*“And then when I’m feeling emotional about it, which is rarer these days… But sometimes you still will just…feel emotional about something that caused you pain and caused your family pain” (#31, F, 18yo, 13.42TSI)*

#### Positive impact of the burn injury on the child or young person

one participant spoke about the resilience they now feel after experiencing a burn injury

*“I guess when I was younger and then now it’s like when I’m feeling stressed, I can acknowledge that I’m feeling stressed and that’s a form of anxiety and it’s gonna be pass, and it’s because of this.” (#31, F, 18yo, 13.42TSI)*

### Factors supporting the recovery journey

#### Coping strategies

participants spoke about strategies they use to cope and what helped during the acute phase of the healing journey. Acceptance and taking a holistic approach were common amongst participants. A few participants mentioned how they now use mindfulness to cope.

*“I think that before I started seeing this psychologist, I actually didn’t deal with stress or anxiety very well, because I didn’t really have the skills to do it. But I think now breathing exercises and stuff like that work.” (#31, F, 18yo, 13.42TSI)*

Leaning on others and social support were another common sub-theme that helped participants cope:

*“I think my close friends are a huge support for me in a whole bunch of different ways. But even in stuff related to my burn, whether it be needing an operation… my friends already supported me through that.” (#31, F, 18yo, 13.42TSI)*

*I mean, whenever I’m with my friends, I’m like, quite happy.” (#22, F, 12yo, 2.17TSI)*

And one participant spoke about the need to positively reframe the injury:

*“I would of like to be able to tell [my story] in a more positive way. Because most of the time, your brain always thinks about the negatives, not the positives.” (#4, F, 13yo, 0.7TSI)*

#### Support services

two sub-themes were developed within support services, the hospital setting and counselling services. One participant mentioned it would have been helpful for the hospital to provide options for parents and families to engage in supports.

*“a more holistic understanding of different options. And for my parents as well, knowing there were options for them as well to get support and for my mum especially, definitely to have more emotional support and [being able to] process her feelings about it.” (#31, F, 18yo, 13.42TSI)*

A few participants talked about accessing counselling during their healing journey, however one participant mentioned they would have liked to have been put in contact with a psychologist earlier on in their recovery journey:

*“And then having the understanding [of] how to reach out for help. And also that it’s the stigma around it as well. I think that it definitely should not have taken me this long to get a psychologist just because it’s just the most normal thing ever. It should be the most normal thing ever” (#31, F, 18yo, 13.42TSI)*

## Discussion

This study aimed to understand the psychosocial impact experienced by pediatric patients following a burn injury. Despite this burn population having a relatively low TBSA percentage (Md = 1.65%) and few days spent in admission (Md = 2 days), our study demonstrated that a burn injury regardless of severity impacts many areas of patients’ lives. Our analysis categorised themes as burn-specific impacts (including appearance concerns, family factors, and lifestyle factors), the psychological impact (including positive and negative impact on mental health), and factors supporting the recovery journey (including coping strategies and support services). The participants in our study highlighted issues they faced during recovery, the positive and negative impacts of the injury and recovery process and provided suggestions for future opportunities to bolster resilience and promote growth for pediatric burn patients who may face similar challenges in the future.

Participants in our study reported on factors that impacted their wellbeing that were specifically outcomes characteristic of a burn injury. One of these factors was appearance concerns, as burn injuries are associated with scarring and having to wear pressure garments during the recovery journey. Participants talked about their burn being a source of anxiety in terms of how they would be perceived by others when they wore a pressure garment and also when the garment was off, and their scar would be seen by others. This theme is consistent with previous research demonstrating that body image concerns in young people contribute to social anxiety, which is likely due to critical periods of social development occurring during adolescence [23, 24]. Often appearance concerns are compounded by lack of education in schools around burn injuries, and certainly in our sample participants were concerned about their peers’ perceptions of them.

Disruption to schooling and other lifestyle factors had a big impact on our participants. Many talked about missing school being a cause of anxiety, as well as missing out on sporting activities. Both sports and schooling can be sources of social support for children and young people, and further, can improve psychological wellbeing [25]. It is not surprising then, that the participants in our study found missing out on both school and sports to be detrimental to their wellbeing.

Recognising caregiver guilt, which often added to the child or young person’s mental health issues, were a consistent theme for the participants in our study. The participants were aware that their caregivers often felt responsible or blamed themselves for the burn injury, and the participants reported that they felt like their caregivers needed more support. This is in line with previous findings that demonstrate caregivers feel guilt relating to their child’s burn injury (Wickens et al., Under Review), which leads to negative mental health issues if left untreated such as anxiety, symptoms of PTSD (such as intrusive thoughts and hypervigilance), and depression [26–29].

In line with previous research, the participants in our study reported heightened anxiety, fear, and posttraumatic stress following their burn injury [5]. The majority (71%) of our participants reported that their mental health was significantly worse after their injury, and of those who reported worse mental health, all stated that they experienced anxiety. This aligns with research showing that children who experience a burn injury are four times more likely to experience anxiety [5]. Worryingly, some participants described symptoms of trauma related stress symptoms, such as nightmares, flashbacks, repetitive thoughts relating to the accident, and hypervigilance. One study has shown that in a sample of toddlers who experienced a burn injury, up to 11.7% met the full criteria for PTSD and 15.3% showed subsyndromal PTSD symptoms [8]. Many of these symptoms reported by the participants in our study were related to the essential procedures that are required for physical recovery (e.g., wound dressings and laser treatments). Again, this is consistent with previous studies on children who have experienced burns, whereby children experience intrusive thoughts related to not only the burn injury itself, but the painful procedures they have to endure during recovery [10].

Interestingly, participants in our study described how they now foster a sense of resilience, in other words they can now acknowledge their negative emotions and process them in a way that allows them to move on. The participants also described helpful coping mechanisms that they have adopted, such as mindfulness, acceptance, and leaning on social support. Indeed, a recent systematic review on the factors that contribute to psychosocial recovery after burn found that social support is the most important factor to promote both resilience after burn for all ages, and posttraumatic growth after burn in adults [15]. Further, another study found that relaxation techniques are useful in relieving procedural anxiety during burn recovery [30]. Trauma-focused therapies often use mindfulness strategies as a therapeutic technique and thus, trauma-focused therapies should be integrated into the recovery process of burn injuries.

Finally, participants were very enthusiastic about mental health support following a burn injury and were very open about seeking help themselves. All participants who sought mental health support described it as being a positive experience that helped them cope immensely. Many participants however, remarked that mental health support should be a part of routine care for patients who experience a burn injury. Further, due to the negative impact that caregivers often feel following their child’s burn [26, 31], evidenced in this study by participants recognising guilt and anxiety felt by their caregivers as well as other family members, a trauma-centred approach for the family unit would be beneficial for children and caregivers who experience a burn. Such interventions or treatments should include resilience building, healthy coping mechanisms and facilitating the expression and regulation of emotions.

## Strengths and limitations

This study aligns with previous research on the impact of pediatric burn injuries and provides evidence that a burn injury impacts many facets of wellbeing however, there are limitations to this study. The generalisability of this study may be impacted by both the relatively small sample size, as well as the fact that this sample is from a single metropolitan area in a Western society. Future studies should endeavour to investigate experiences of children and young people from rural and remote areas. Although data saturation is a commonly employed practice to evaluate data collection, it is inconsistent with reflexive thematic analysis by Braun and Clarke [22, 32]. Despite this, children were recruited until no new relevant knowledge was obtained concerning the topics of the psychosocial impact of pediatric burns. Another limitation was the variability in time since the burn injury, which may place the child or young person at risk of recall bias. In addition, one participant requested to have their mother present during their interview. Although the child felt more comfortable having their parent there, it could have limited the child’s willingness to be open with their responses. It is also important to note that this study did not include a clinical evaluation of mental health conditions, and future research should employ a mixed methods approach to this area of research. Although we must consider this study within the context of these limitations, this study nonetheless provides valuable insight into the experiences of children and young people following a burn injury, from their own perspective, which can be used to help inform future work and approaches to support recovery.

## Implications for research, policy and practice

This study has shown that children and young people who experience a burn face many hardships, which should be acknowledged by clinical care teams in the recovery journey. Factors that improve the mental health and wellbeing of children and young people who sustain a burn injury should be promoted, such as mental health and social supports, the promotion of adaptive coping mechanisms, and meeting the needs of the family as a whole. To improve the transition back into school, hospitals and clinical care teams could work with schools to prepare and educate staff and students on burn injuries. Additionally, programs and systems of care should prioritise using a trauma-informed approach for pediatric burn patients, which includes recognising the signs and symptoms of trauma, and understanding the patients’ vulnerabilities and triggers, to avoid re-traumatisation. It is then important to validate their individual experience and tailor treatment plans that focus on trauma healing. Ultimately, the implementation of trauma-focused, family centred interventions is crucial for the psychosocial recovery of pediatric burn survivors.

## Electronic supplementary material

Below is the link to the electronic supplementary material.

Supplementary Material 1

Supplementary Material 2

## Data Availability

The datasets generated during and analysed during the current study are not publicly available due to the sensitivity of participant data but are available from the corresponding author on reasonable request. Every request will be reviewed by the institutional review board at the Telethon Kids Institute; the researcher will need to sign a data access agreement with the Telethon Kids Institute after approval.
