# Supplementary material for: “I just get scared it’s going to happen again”: a qualitative study of the psychosocial impact of pediatric burns from the child’s perspective
Source: BMC Pediatr. 2023 Jun 5;23:280. doi: 10.1186/s12887-023-04105-y (PMC10240749; doi:10.1186/s12887-023-04105-y)
Supplement: Supplementary file 1 — Supplementary Material 1 [file 12887_2023_4105_MOESM1_ESM.docx]

**3.2 Burn-specific impact on the child or young person**

**Family factors:**

Immediate Family

*“Because obviously, there is a lot of guilt attached for parents, I think when like, your kid has an accident. And then like your parent has to like when you're really little, and your parent has to question, oh gosh, like what if I was watching her? What if I was like, you know, all of these things? And like, so I think it was, it's equally as hard for like parents of young kids that experience burns. But um, yeah, so I think like a big part of that was like, her getting that kind of anxiety from that” (FG9)*

**Lifestyle factors:**

Sports

*“I wasn't actually able to do that with all the bandages. I kind of over we would just stop. But then it was also like, I kind of overthink it again. I was just like, oh, I'm in this, I won't be able to do this or something” (FG1)*

*“Well, because most of my friends, well, most of my close friends played in my netball team, and all that type of stuff. And I had to miss out on half the season. So I was, I would still go wait and watch, but it still didn't feel the same as actually playing it” (FG5)*

School

*“I reckon I just, I kind of felt that I was behind. And I wasn't able to do like, what I like all my work, and I was kind of behind in everyone else. And then I'm just like, I had projects, I had one in science and I like even I tell the teacher that I can't actually, I couldn't do it because I was in hospital, he would still say, oh, that's no excuse. So he would still make me do it and so it's just more added pressure” (FG1)*

- 1. **Psychological impact of the burn injury on the child or young person**

**Negative impact on mental health:**

Catastrophising

*“While in hospital and a while afterwards I couldn't stop thinking about how else it could go wrong or worse, like if I accidentally set the house on fire and killed my whole family” (FG16)*

*“I mean at first I was like really worried about like what was going to happen. And like, just all the possible outcomes… I really just thought like, about the worst possible scenario” (FG12)*

Hypervigilance

*“And being like, hyper aware of like, I'm not good with car accidents I'm like, terrified of like, I get very nervous about driving” (#31, F, 18yo, 13.42TSI)*

*“Yeah, I'm probably more cautious because the doctors told me like, the scars still. Like, you can still tear or like, it's fragile, fragile. And I haven't like really wanted don't really want it to break again, or like, so I've been a bit cautious of that” (FG11)*

Hallucination

*“I had one really short hallucination at school where it looked like the book i was using was charred… I haven't had any kind of hallucination before” (FG16)*

- 1. **Factors supporting the recovery journey**

**Coping strategies**

Mindfulness

*“I would kind of just get more nervous about taking off the garment and stuff…there wasn't really much that I did for myself to calm myself down. But I reckon if I like had, if I was very upset, I usually do some type of mindfulness, or I just say, take breaths, or something” (FG1)*
