# Supplementary material for: “I just get scared it’s going to happen again”: a qualitative study of the psychosocial impact of pediatric burns from the child’s perspective
Source: BMC Pediatr. 2023 Jun 5;23:280. doi: 10.1186/s12887-023-04105-y (PMC10240749; doi:10.1186/s12887-023-04105-y)
Supplement: Supplementary file 2 — Supplementary Material 2 [file 12887_2023_4105_MOESM2_ESM.docx]

**Appendix 1: Interview guide and semi-structured interview schedule**

**Interviewer Guidelines**

1. **Welcome** - includes Acknowledgement of Country, permission to record, confidentiality and general guidelines, which included the ability to decline answering questions or withdrawing from interview.
2. **Introduction** - includes interviewer’s professional background and role in the project.
3. **Project information/purpose** – an explanation for the aim of the interview; overview and feedback of a novel program developed by team; time for questions.

**Semi-structure interview schedule**

1. How has your mental health and wellbeing been since your injury?
2. Is there anything that has concerned you about your mental health after your injury?
   1. If yes: Which of these is bothering you the most?
3. Are there any problems that you think have gotten worse since your injury?
4. Have you felt overwhelmed by stress since your injury?
5. How do you calm yourself down when you’re stressed? Does it work?
6. What did you enjoy doing before your injury? Do you still enjoy these activities?
7. Is your family getting support from outside sources, or are you coping on your own as a family?
8. Have you felt the need to reach out to friends or family to help after your injury?
9. Do you think your family would benefit from a list of sources available to help cope with your injury?
   1. (If yes: Provide with support services)

How have you been doing at school since your injury?

Helpful prompts :

“With that information, let me re-ask the question”

“You already alluded to x”- move into next question

“Do you have something more to add?”

“Might pull it back to some questions we have here”

Note: the authors do not wish to share the raw data from this study as we 1) do not have ethics approval to do so and, 2) our data is not de-identifiable in it’s raw form.
